# 4-Carbamoylpyridin-1-ium 2,2,2-tri­chloro­acetate–isonicotinamide (1/1)

**DOI:** 10.1107/S1600536812037002

**Published:** 2012-08-31

**Authors:** Franc Perdih

**Affiliations:** a Faculty of Chemistry and Chemical Technology, University of Ljubljana, Aškerčeva 5, PO Box 537, SI-1000 Ljubljana, Slovenia; b CO EN–FIST, Dunajska 156, SI-1000 Ljubljana, Slovenia

## Abstract

In the crystal structure of the title 1:1 co-crystal, C_6_H_7_N_2_O^+^·C_2_Cl_3_O_2_^−^·C_6_H_6_N_2_O, the amide groups of the 4-carbamoylpyridin-1-ium ion and the isonicotinamide mol­ecule are twisted out of the plane of the aromatic ring with C—C—C—N torsion angles of 21.5 (4) and −33.5 (4)°, respectively. The 4-carbamoylpyridin-1-ium and isonicotinamide amide groups form *R*_2_^2^(8) hydrogen-bonded dimers *via* N—H⋯O=C inter­actions. The two remaining amide H atoms (i) link dimers *via* the cation to an isonicotinamide and (ii) from the isonicotinamide to a trichloro­acetate anion. The pyridinium H atom also forms an N—H⋯O hydrogen bond with the trichloro­acetate anion. Due to the extended hydrogen bonding, including C—H⋯O and C—H⋯Cl interactions, all components in the structure aggregate into a three-dimensional supra­molecular framework.

## Related literature

For applications of co-crystals, see: Karki *et al.* (2009 ▶); Friščić & Jones (2010 ▶). For related structures, see: Madeley *et al.* (2011 ▶).
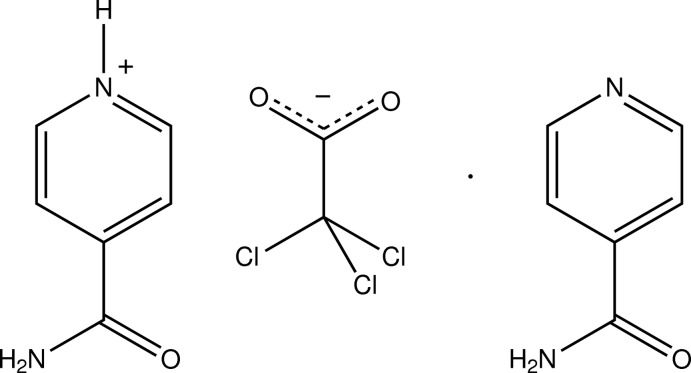


## Experimental

### 

#### Crystal data

C_6_H_7_N_2_O^+^·C_2_Cl_3_O_2_^−^·C_6_H_6_N_2_O
*M* *_r_* = 407.63
Orthorhombic, 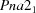

*a* = 13.7910 (3) Å
*b* = 22.6680 (5) Å
*c* = 5.6340 (1) Å
*V* = 1761.27 (6) Å^3^
*Z* = 4
Mo *K*α radiation
μ = 0.55 mm^−1^
*T* = 293 K
0.4 × 0.1 × 0.1 mm

#### Data collection

Agilent SuperNova, Dual, Cu at zero, Atlas diffractometer
Absorption correction: multi-scan (*CrysAlis PRO*; Agilent, 2011 ▶) *T*_min_ = 0.811, *T*_max_ = 0.947
16297 measured reflections
4017 independent reflections
3575 reflections with *I* > 2σ(*I*)
*R*_int_ = 0.031

#### Refinement

*R*[*F* ^2^ > 2σ(*F* ^2^)] = 0.04
*wR*(*F* ^2^) = 0.091
*S* = 1.04
4017 reflections
241 parameters
1 restraint
H atoms treated by a mixture of independent and constrained refinement
Δρ_max_ = 0.41 e Å^−3^
Δρ_min_ = −0.57 e Å^−3^
Absolute structure: Flack (1983) ▶, 1791 Friedel pairs
Flack parameter: 0.01 (6)

### 

Data collection: *CrysAlis PRO* (Agilent, 2011 ▶); cell refinement: *CrysAlis PRO*; data reduction: *CrysAlis PRO*; program(s) used to solve structure: *SHELXS97* (Sheldrick, 2008 ▶); program(s) used to refine structure: *SHELXL97* (Sheldrick, 2008 ▶); molecular graphics: *ORTEP-3 for Windows* (Farrugia, 1997 ▶) and *DIAMOND* (Brandenburg, 1999 ▶); software used to prepare material for publication: *WinGX* (Farrugia, 1999 ▶) and *publCIF* (Westrip, 2010 ▶).

## Supplementary Material

Crystal structure: contains datablock(s) global, I. DOI: 10.1107/S1600536812037002/gg2095sup1.cif

Structure factors: contains datablock(s) I. DOI: 10.1107/S1600536812037002/gg2095Isup2.hkl

Supplementary material file. DOI: 10.1107/S1600536812037002/gg2095Isup3.cml

Additional supplementary materials:  crystallographic information; 3D view; checkCIF report

## Figures and Tables

**Table 1:** Hydrogen-bond geometry (Å, °)

*D*—H⋯*A*	*D*—H	H⋯*A*	*D*⋯*A*	*D*—H⋯*A*
N1—H15⋯O2^i^	0.90 (3)	1.78 (3)	2.679 (3)	175 (3)
N2—H16*A*⋯N3^ii^	0.87 (3)	2.11 (3)	2.958 (3)	164 (3)
N2—H16*B*⋯O3	0.90 (4)	1.99 (4)	2.887 (3)	178 (3)
N4—H17*A*⋯O4	0.91 (4)	2.08 (4)	2.972 (3)	167 (4)
N4—H17*B*⋯O1	0.89 (4)	1.98 (4)	2.839 (3)	160 (3)
C1—H1⋯O1^i^	0.93	2.58	3.211 (3)	126
C2—H2⋯O4^iii^	0.93	2.55	3.358 (3)	146
C7—H7⋯O3^iv^	0.93	2.58	3.489 (3)	166
C11—H11⋯Cl2^v^	0.93	2.82	3.711 (3)	162

Symmetry codes: (i) 
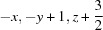
; (ii) 
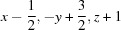
; (iii) 
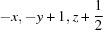
; (iv) 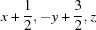; (v) 
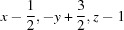
.

**Fig. 1.**
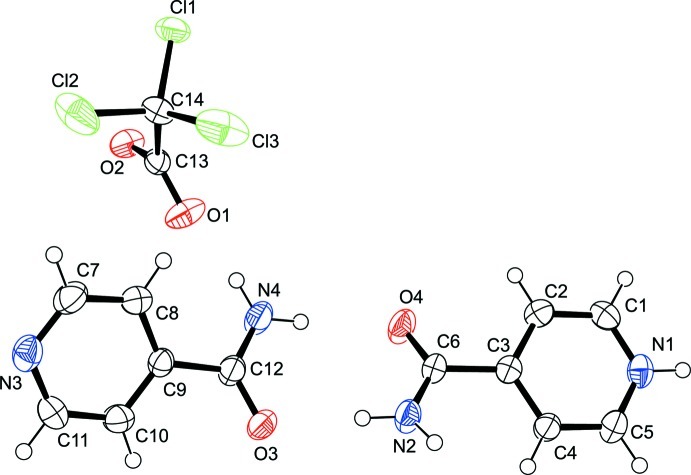
The asymmetric unit of the title compound with displacement ellipsoids drawn at the 50% probability level.

**Fig. 2.**
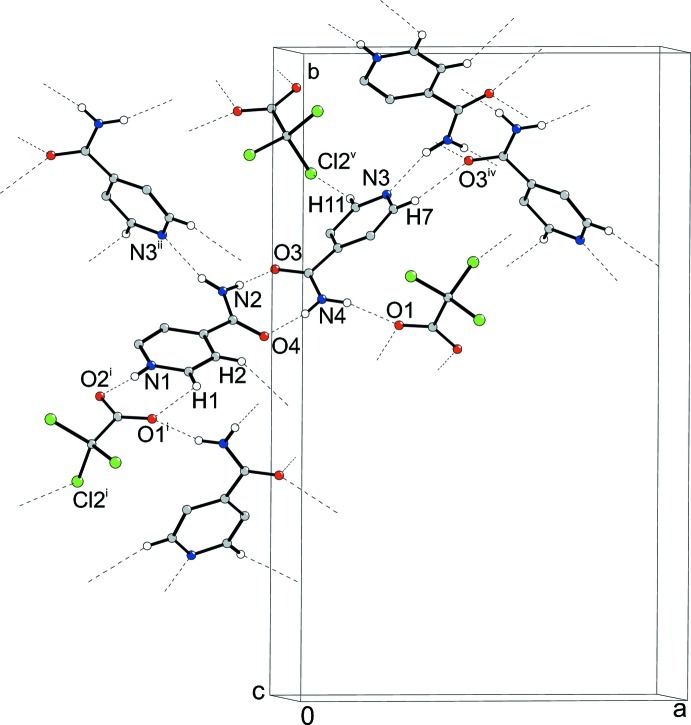
Hydrogen bonding diagram. Dashed lines indicate intermolecular N—H···O, N—H···N, C—H···O and C—H···Cl hydrogen bonding. For the sake of clarity, H atoms not involved in the motif shown have been omitted. Symmetry codes: i –x, –y + 1, z + 3/2; iix – 1/2, –y + 3/2, z + 1; ivx + 1/2, –y + 3/2, z; vx – 1/2, –y + 3/2, z – 1.

**Fig. 3.**
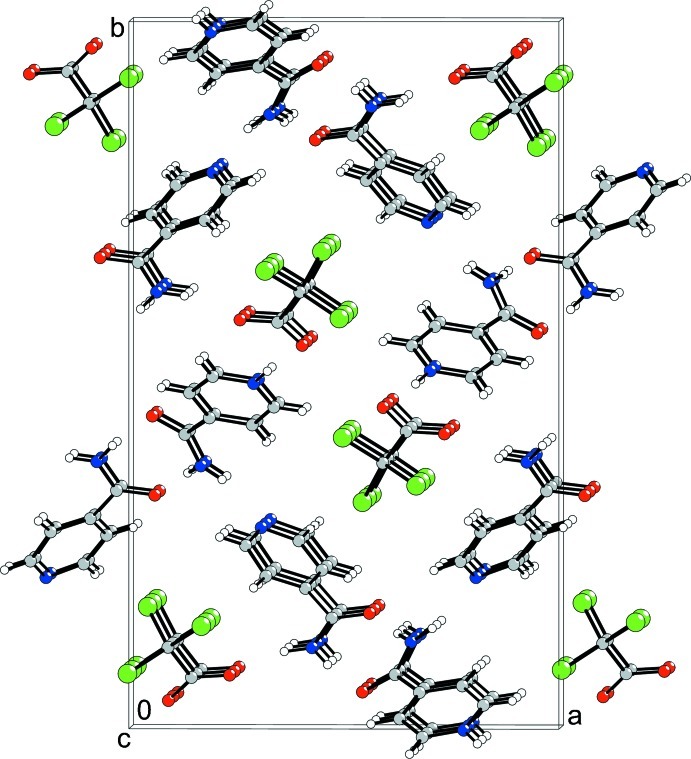
Crystal packing of the title compound. For the sake of clarity, hydrogen bonding is not presented.

## supplementary crystallographic information

### Comment

Co-crystals have attracted much attention in recent years owing to their contributions to crystal engineering and pharmaceutical chemistry. They were found to be useful in improving the stability, solubility, dissolution rate and mechanical properties (Karki *et al.*, 2009; Friščić & Jones, 2010). Here we present the structure obtained by reacting isonicotinamide and trichloroacetic acid in 2:1 molar ratio.

The asymmetric unit of (I) consists of one 4-carbamoylpyridin-1-ium cation, one trichloroacetate anion and one isonicotinamide molecule (Fig. 1). The amide groups of 4-carbamoylpyridin-1-ium ion and isonicotinamide molecule are twisted out of the plane of the aromatic ring with a C—C—C—N torsion angle of 21.5 (4)° and -33.5 (4)°, respectively. Similar twisting was observed for example in isonicotinamide–2-naphthoic acid (1/1) (Madeley *et al.*, 2011). Aromatic rings of 4-carbamoylpyridin-1-ium ion and isonicotinamide molecule are not coplanar, but are inclined by 35.05 (12)°. In the crystal, all the components of the structure are associated *via* the extended system of hydrogen bonds (N—H···O and N—H···N) and weak C—H···O and C—H···Cl interactions into extended three-dimensional supramolecular framework (Figs. 2, 3). The 4-carbamoylpyridin-1-ium ion is hydrogen bonded *via* N—H···O hydrogen bonding of the pyridinium unit to the trichloroacetate ion. The amide groups from 4-carbamoylpyridin-1-ium and isonicotinamide form a dimer *via* N—H···O hydrogen bonding, that is a typical supramolecular hydrogen-bonded synthon observed for amide-amide homodimers. Furthermore, the amide group of the cation is hydrogen bonded to the pyridine unit of isonicotinamide and the amide group of the isonicotinamide is hydrogen bonded to the trichloroacetate ion.

### Experimental

Crystals of the title compound were obtained by slow evaporation of a 2:1 mol. mixture of isonicotinamide and trichloroacetic acid in methanol at room temperature.

### Refinement

All H atoms were initially located in a difference Fourier maps. H atoms attached to N atoms were refined isotropically with *U*_iso_(H) = 1.5*U*_eq_(N). Other H atoms were treated as riding atoms in geometrically idealized positions, with C—H = 0.93 Å, and with *U*_iso_(H) = 1.2*U*_eq_(C).

### Crystal data

**Table:**

C_6_H_7_N_2_O^+^·C_2_Cl_3_O_2_^−^·C_6_H_6_N_2_O	*F*(000) = 832
*M**_r_* = 407.63	*D*_x_ = 1.537 Mg m^−^^3^
Orthorhombic, *P**n**a*2_1_	Mo *K*α radiation, λ = 0.71073 Å
Hall symbol: P 2c -2n	Cell parameters from 7898 reflections
*a* = 13.7910 (3) Å	θ = 3.1–30.4°
*b* = 22.6680 (5) Å	µ = 0.55 mm^−^^1^
*c* = 5.6340 (1) Å	*T* = 293 K
*V* = 1761.27 (6) Å^3^	Prism, colourless
*Z* = 4	0.4 × 0.1 × 0.1 mm

### Data collection

**Table:**

Agilent SuperNova, Dual, Cu at zero, Atlas diffractometer	4017 independent reflections
Radiation source: SuperNova (Mo) X-ray Source	3575 reflections with *I* > 2σ(*I*)
Mirror monochromator	*R*_int_ = 0.031
Detector resolution: 10.4933 pixels mm^-1^	θ_max_ = 27.5°, θ_min_ = 3.1°
ω scans	*h* = −17→17
Absorption correction: multi-scan (*CrysAlis PRO*; Agilent, 2011)	*k* = −29→29
*T*_min_ = 0.811, *T*_max_ = 0.947	*l* = −7→7
16297 measured reflections	

### Refinement

**Table:**

Refinement on *F*^2^	Secondary atom site location: difference Fourier map
Least-squares matrix: full	Hydrogen site location: inferred from neighbouring sites
*R*[*F*^2^ > 2σ(*F*^2^)] = 0.04	H atoms treated by a mixture of independent and constrained refinement
*wR*(*F*^2^) = 0.091	*w* = 1/[σ^2^(*F*_o_^2^) + (0.0359*P*)^2^ + 0.8943*P*] where *P* = (*F*_o_^2^ + 2*F*_c_^2^)/3
*S* = 1.04	(Δ/σ)_max_ < 0.001
4017 reflections	Δρ_max_ = 0.41 e Å^−^^3^
241 parameters	Δρ_min_ = −0.57 e Å^−^^3^
1 restraint	Absolute structure: Flack (1983), 1791 Friedel pairs
Primary atom site location: structure-invariant direct methods	Flack parameter: 0.01 (6)

### Special details

**Table:**

Geometry. All s.u.'s (except the s.u. in the dihedral angle between two l.s. planes) are estimated using the full covariance matrix. The cell s.u.'s are taken into account individually in the estimation of s.u.'s in distances, angles and torsion angles; correlations between s.u.'s in cell parameters are only used when they are defined by crystal symmetry. An approximate (isotropic) treatment of cell s.u.'s is used for estimating s.u.'s involving l.s. planes.
Refinement. Refinement of *F*^2^ against ALL reflections. The weighted *R*-factor *wR* and goodness of fit *S* are based on *F*^2^, conventional *R*-factors *R* are based on *F*, with *F* set to zero for negative *F*^2^. The threshold expression of *F*^2^ > 2σ(*F*^2^) is used only for calculating *R*-factors(gt) *etc*. and is not relevant to the choice of reflections for refinement. *R*-factors based on *F*^2^ are statistically about twice as large as those based on *F*, and *R*- factors based on ALL data will be even larger.

### Fractional atomic coordinates and isotropic or equivalent isotropic displacement parameters (Å^2^)

**Table:**

	*x*	*y*	*z*	*U*_iso_*/*U*_eq_	
Cl1	0.49348 (5)	0.58322 (3)	0.45563 (14)	0.04547 (17)	
Cl2	0.44990 (9)	0.67585 (4)	0.11741 (18)	0.0850 (4)	
Cl3	0.32105 (7)	0.65069 (5)	0.50880 (15)	0.0770 (3)	
N1	−0.29717 (16)	0.50785 (9)	1.1681 (4)	0.0377 (5)	
H15	−0.330 (2)	0.4892 (15)	1.285 (6)	0.057*	
N2	−0.16743 (16)	0.62843 (10)	0.4929 (5)	0.0396 (5)	
H16A	−0.217 (2)	0.6478 (14)	0.551 (7)	0.059*	
H16B	−0.135 (3)	0.6394 (15)	0.362 (6)	0.059*	
N3	0.19060 (17)	0.78523 (10)	−0.3220 (5)	0.0425 (5)	
N4	0.06680 (19)	0.61873 (11)	0.1998 (5)	0.0495 (7)	
H17A	0.030 (3)	0.5952 (17)	0.294 (8)	0.074*	
H17B	0.131 (3)	0.6133 (15)	0.197 (7)	0.074*	
O1	0.25905 (13)	0.57929 (9)	0.1146 (4)	0.0524 (5)	
O2	0.40234 (13)	0.54301 (8)	0.0115 (4)	0.0426 (4)	
O3	−0.06660 (12)	0.66646 (9)	0.0707 (4)	0.0473 (5)	
O4	−0.05512 (13)	0.55797 (8)	0.5595 (4)	0.0488 (5)	
C1	−0.20142 (19)	0.49801 (11)	1.1584 (5)	0.0384 (6)	
H1	−0.1721	0.4742	1.2722	0.046*	
C2	−0.14636 (17)	0.52287 (10)	0.9812 (5)	0.0356 (5)	
H2	−0.0801	0.5155	0.9729	0.043*	
C3	−0.19079 (17)	0.55906 (10)	0.8150 (4)	0.0293 (5)	
C4	−0.29018 (18)	0.56864 (11)	0.8322 (5)	0.0335 (5)	
H4	−0.3214	0.5929	0.7233	0.04*	
C5	−0.34193 (18)	0.54211 (11)	1.0107 (5)	0.0387 (6)	
H5	−0.4085	0.5481	1.0217	0.046*	
C6	−0.13130 (17)	0.58311 (10)	0.6111 (5)	0.0320 (5)	
C7	0.2246 (2)	0.76108 (12)	−0.1226 (6)	0.0454 (7)	
H7	0.2848	0.7735	−0.0675	0.055*	
C8	0.17586 (17)	0.71890 (11)	0.0064 (5)	0.0396 (6)	
H8	0.2034	0.7027	0.1424	0.048*	
C9	0.08482 (16)	0.70091 (10)	−0.0701 (5)	0.0305 (5)	
C10	0.0493 (2)	0.72548 (12)	−0.2753 (5)	0.0384 (6)	
H10	−0.0114	0.7145	−0.3326	0.046*	
C11	0.1043 (2)	0.76648 (11)	−0.3955 (5)	0.0433 (6)	
H11	0.0797	0.7819	−0.5361	0.052*	
C12	0.02224 (18)	0.65981 (11)	0.0724 (5)	0.0358 (6)	
C13	0.34726 (17)	0.57605 (10)	0.1255 (4)	0.0306 (5)	
C14	0.40010 (19)	0.61971 (11)	0.2972 (5)	0.0368 (6)	

### Atomic displacement parameters (Å^2^)

**Table:**

	*U*^11^	*U*^22^	*U*^33^	*U*^12^	*U*^13^	*U*^23^
Cl1	0.0384 (3)	0.0586 (4)	0.0395 (3)	0.0069 (3)	−0.0118 (3)	0.0020 (3)
Cl2	0.1224 (9)	0.0644 (5)	0.0681 (6)	−0.0526 (6)	−0.0447 (6)	0.0302 (5)
Cl3	0.0848 (6)	0.0950 (6)	0.0510 (5)	0.0508 (5)	−0.0198 (4)	−0.0362 (5)
N1	0.0414 (12)	0.0353 (11)	0.0365 (13)	−0.0078 (9)	0.0084 (10)	0.0028 (9)
N2	0.0375 (12)	0.0379 (11)	0.0435 (13)	0.0082 (9)	0.0147 (11)	0.0077 (11)
N3	0.0423 (13)	0.0351 (11)	0.0503 (14)	−0.0051 (9)	0.0080 (11)	0.0057 (10)
N4	0.0317 (12)	0.0490 (14)	0.0678 (17)	0.0069 (10)	0.0141 (12)	0.0256 (13)
O1	0.0277 (9)	0.0589 (12)	0.0706 (14)	0.0023 (8)	−0.0027 (10)	−0.0192 (12)
O2	0.0346 (9)	0.0506 (10)	0.0426 (10)	0.0042 (8)	−0.0017 (8)	−0.0179 (9)
O3	0.0278 (9)	0.0557 (11)	0.0585 (13)	0.0039 (8)	0.0068 (9)	0.0201 (10)
O4	0.0367 (10)	0.0514 (11)	0.0585 (14)	0.0153 (8)	0.0200 (9)	0.0148 (10)
C1	0.0444 (15)	0.0377 (13)	0.0332 (15)	0.0002 (11)	−0.0051 (11)	0.0055 (11)
C2	0.0307 (11)	0.0348 (12)	0.0413 (14)	0.0014 (9)	0.0001 (12)	0.0002 (12)
C3	0.0297 (12)	0.0279 (11)	0.0304 (12)	−0.0023 (9)	0.0021 (10)	−0.0034 (9)
C4	0.0300 (13)	0.0340 (13)	0.0364 (13)	0.0012 (10)	0.0031 (11)	0.0044 (10)
C5	0.0347 (13)	0.0381 (13)	0.0435 (14)	−0.0010 (10)	0.0099 (12)	0.0013 (12)
C6	0.0295 (12)	0.0343 (12)	0.0321 (12)	−0.0013 (9)	0.0071 (10)	−0.0003 (11)
C7	0.0327 (14)	0.0460 (16)	0.0577 (18)	−0.0081 (12)	−0.0007 (12)	0.0027 (14)
C8	0.0317 (12)	0.0450 (14)	0.0422 (15)	0.0006 (10)	−0.0029 (11)	0.0061 (13)
C9	0.0288 (11)	0.0289 (11)	0.0337 (12)	0.0018 (9)	0.0050 (10)	0.0020 (10)
C10	0.0337 (13)	0.0412 (14)	0.0404 (14)	−0.0027 (11)	−0.0053 (11)	0.0025 (12)
C11	0.0490 (15)	0.0441 (14)	0.0369 (14)	0.0005 (12)	−0.0016 (13)	0.0113 (13)
C12	0.0313 (13)	0.0343 (12)	0.0418 (15)	0.0018 (10)	0.0064 (11)	0.0060 (11)
C13	0.0323 (12)	0.0325 (11)	0.0269 (11)	−0.0016 (9)	−0.0010 (10)	−0.0008 (10)
C14	0.0435 (15)	0.0342 (14)	0.0327 (12)	0.0039 (11)	−0.0083 (11)	−0.0023 (11)

### Geometric parameters (Å, º)

**Table:**

Cl1—C14	1.772 (3)	C1—H1	0.93
Cl2—C14	1.765 (3)	C2—C3	1.387 (4)
Cl3—C14	1.762 (3)	C2—H2	0.93
N1—C5	1.331 (4)	C3—C4	1.391 (3)
N1—C1	1.340 (3)	C3—C6	1.513 (3)
N1—H15	0.90 (3)	C4—C5	1.372 (4)
N2—C6	1.322 (3)	C4—H4	0.93
N2—H16A	0.87 (3)	C5—H5	0.93
N2—H16B	0.90 (4)	C7—C8	1.376 (4)
N3—C11	1.330 (4)	C7—H7	0.93
N3—C7	1.335 (4)	C8—C9	1.389 (3)
N4—C12	1.326 (3)	C8—H8	0.93
N4—H17A	0.91 (4)	C9—C10	1.374 (4)
N4—H17B	0.89 (4)	C9—C12	1.503 (3)
O1—C13	1.220 (3)	C10—C11	1.378 (4)
O2—C13	1.245 (3)	C10—H10	0.93
O3—C12	1.234 (3)	C11—H11	0.93
O4—C6	1.230 (3)	C13—C14	1.564 (3)
C1—C2	1.375 (4)		
			
C5—N1—C1	121.8 (2)	N3—C7—C8	123.9 (3)
C5—N1—H15	122 (2)	N3—C7—H7	118
C1—N1—H15	116 (2)	C8—C7—H7	118
C6—N2—H16A	120 (2)	C7—C8—C9	118.8 (3)
C6—N2—H16B	116 (2)	C7—C8—H8	120.6
H16A—N2—H16B	124 (3)	C9—C8—H8	120.6
C11—N3—C7	116.4 (2)	C10—C9—C8	117.7 (2)
C12—N4—H17A	118 (2)	C10—C9—C12	119.7 (2)
C12—N4—H17B	123 (2)	C8—C9—C12	122.4 (2)
H17A—N4—H17B	119 (3)	C9—C10—C11	119.4 (2)
N1—C1—C2	120.3 (2)	C9—C10—H10	120.3
N1—C1—H1	119.8	C11—C10—H10	120.3
C2—C1—H1	119.8	N3—C11—C10	123.7 (3)
C1—C2—C3	119.2 (2)	N3—C11—H11	118.1
C1—C2—H2	120.4	C10—C11—H11	118.1
C3—C2—H2	120.4	O3—C12—N4	123.4 (2)
C2—C3—C4	118.7 (2)	O3—C12—C9	119.3 (2)
C2—C3—C6	119.1 (2)	N4—C12—C9	117.3 (2)
C4—C3—C6	122.1 (2)	O1—C13—O2	128.2 (2)
C5—C4—C3	119.7 (2)	O1—C13—C14	117.2 (2)
C5—C4—H4	120.2	O2—C13—C14	114.6 (2)
C3—C4—H4	120.2	C13—C14—Cl3	112.49 (18)
N1—C5—C4	120.2 (2)	C13—C14—Cl2	106.43 (18)
N1—C5—H5	119.9	Cl3—C14—Cl2	109.97 (15)
C4—C5—H5	119.9	C13—C14—Cl1	110.78 (17)
O4—C6—N2	124.3 (2)	Cl3—C14—Cl1	107.15 (15)
O4—C6—C3	118.4 (2)	Cl2—C14—Cl1	110.04 (15)
N2—C6—C3	117.3 (2)		
			
C5—N1—C1—C2	−0.8 (4)	C7—C8—C9—C12	−173.6 (2)
N1—C1—C2—C3	1.1 (4)	C8—C9—C10—C11	−0.1 (4)
C1—C2—C3—C4	−0.5 (4)	C12—C9—C10—C11	175.2 (2)
C1—C2—C3—C6	−176.0 (2)	C7—N3—C11—C10	1.5 (4)
C2—C3—C4—C5	−0.4 (4)	C9—C10—C11—N3	−1.5 (4)
C6—C3—C4—C5	175.0 (2)	C10—C9—C12—O3	−30.3 (4)
C1—N1—C5—C4	−0.1 (4)	C8—C9—C12—O3	144.8 (3)
C3—C4—C5—N1	0.7 (4)	C10—C9—C12—N4	151.4 (3)
C2—C3—C6—O4	20.0 (4)	C8—C9—C12—N4	−33.5 (4)
C4—C3—C6—O4	−155.4 (3)	O1—C13—C14—Cl3	17.9 (3)
C2—C3—C6—N2	−163.1 (2)	O2—C13—C14—Cl3	−163.93 (19)
C4—C3—C6—N2	21.5 (4)	O1—C13—C14—Cl2	−102.6 (3)
C11—N3—C7—C8	0.1 (4)	O2—C13—C14—Cl2	75.6 (2)
N3—C7—C8—C9	−1.6 (4)	O1—C13—C14—Cl1	137.8 (2)
C7—C8—C9—C10	1.5 (4)	O2—C13—C14—Cl1	−44.0 (3)

### Hydrogen-bond geometry (Å, º)

**Table:**

*D*—H···*A*	*D*—H	H···*A*	*D*···*A*	*D*—H···*A*
N1—H15···O2^i^	0.90 (3)	1.78 (3)	2.679 (3)	175 (3)
N2—H16*A*···N3^ii^	0.87 (3)	2.11 (3)	2.958 (3)	164 (3)
N2—H16*B*···O3	0.90 (4)	1.99 (4)	2.887 (3)	178 (3)
N4—H17*A*···O4	0.91 (4)	2.08 (4)	2.972 (3)	167 (4)
N4—H17*B*···O1	0.89 (4)	1.98 (4)	2.839 (3)	160 (3)
C1—H1···O1^i^	0.93	2.58	3.211 (3)	126
C2—H2···O4^iii^	0.93	2.55	3.358 (3)	146
C7—H7···O3^iv^	0.93	2.58	3.489 (3)	166
C11—H11···Cl2^v^	0.93	2.82	3.711 (3)	162

Symmetry codes: (i) −*x*, −*y*+1, *z*+3/2; (ii) *x*−1/2, −*y*+3/2, *z*+1; (iii) −*x*, −*y*+1, *z*+1/2; (iv) *x*+1/2, −*y*+3/2, *z*; (v) *x*−1/2, −*y*+3/2, *z*−1.
